# Prevalence and correlates of vitamin D deficiency in adults after traumatic brain injury

**DOI:** 10.1111/cen.13045

**Published:** 2016-03-28

**Authors:** Omer A. Jamall, Claire Feeney, Joanna Zaw‐Linn, Aysha Malik, Mari E.K. Niemi, Carmen Tenorio‐Jimenez, Timothy E. Ham, Sagar R. Jilka, Peter O. Jenkins, Gregory Scott, Lucia M. Li, Nikolaos Gorgoraptis, David Baxter, David J. Sharp, Anthony P. Goldstone

**Affiliations:** ^1^ Computational, Cognitive and Clinical Neuroimaging LaboratoryDivision of Brain SciencesImperial College LondonHammersmith HospitalLondonUK; ^2^ Imperial Centre for EndocrinologyImperial College Healthcare NHS TrustSt. Mary's HospitalLondonUK; ^3^ Centre for NeuropsychopharmacologyDivision of Brain SciencesImperial College LondonHammersmith HospitalLondonUK

## Abstract

**Objectives:**

Traumatic brain injury (TBI) is a major cause of long‐term disability with variable recovery. Preclinical studies suggest that vitamin D status influences the recovery after TBI. However, there is no published clinical data on links between vitamin D status and TBI outcomes. The aim was to determine the (i) prevalence of vitamin D deficiency/insufficiency, and associations of vitamin D status with (ii) demographic factors and TBI severity, and with (iii) cognitive function, symptoms and quality of life, in adults after TBI.

**Design:**

Retrospective audit of patients seen between July 2009 and March 2015. Serum vitamin D (25‐hydroxy‐cholecalciferol) was categorized as deficient (<40 nmol/l), insufficient (40–70 nmol/l) or replete (>70 nmol/l).

**Patients:**

A total of 353 adults seen in tertiary hospital clinic (75·4% lighter skinned, 74·8% male, age median 35·1 year, range 26·6–48·3 year), 0·3–56·5 months after TBI (74·5% moderate–severe).

**Measurements:**

Serum vitamin D concentrations; Addenbrooke's Cognitive Examination (ACE‐R), Beck Depression Inventory‐II (BDI‐II), SF‐36 Quality of Life, Pittsburgh Sleep Quality Index.

**Results:**

In total, 46·5% of patients after TBI had vitamin D deficiency and 80·2% insufficiency/deficiency. Patients with vitamin D deficiency had lower ACE‐R scores than those of vitamin D replete (mean effect size ± SEM 4·5 ± 2·1, *P* = 0·034), and higher BDI‐II scores than those of vitamin D insufficient (4·5 ± 1·6, *P* = 0·003), correcting for age, gender, time since TBI and TBI severity. There was no association between vitamin D status and markers of TBI severity, sleep or quality of life.

**Conclusion:**

Vitamin D deficiency is common in patients after TBI and associated with impaired cognitive function and more severe depressive symptoms.

## Introduction

Each year, 1·4 million people attend emergency departments in England and Wales with a recent traumatic brain injury (TBI).^1^ TBI is a major cause of long‐term disability often leading to neuropsychiatric and cognitive impairments, including problems with memory and executive function, mood, sleep disturbance and lethargy. Recovery after TBI varies markedly between patients. Neuroendocrine dysfunction leading to pituitary hormone deficiencies after TBI may contribute to persistent symptoms, especially growth hormone deficiency, with 5–20% of TBI patients reported as having hypothalamic–pituitary dysfunction.^2^ Vitamin D is another hormonal factor that could influence recovery after TBI. Vitamin D is a fat‐soluble sercosteroid essential for musculoskeletal health that is primarily synthesized in the skin upon sun exposure. The prevalence of vitamin D deficiency may be increased after TBI because of reduced sun exposure as a result of hospitalization, impaired social functioning and absence from work, resulting in more time spent indoors.

Vitamin D deficiency has been associated with many systemic conditions, such as obesity, cardiovascular and neurodegenerative diseases.^3^, ^4^ Several meta‐analyses have linked vitamin D status to depression 5, 6 (Table S2) and impaired cognitive function.^7^, ^8^, ^9^, ^10^ However, these clinical findings are from association studies and so it is difficult to infer causation as any disorder that decreases exposure to sunlight, such as depression, may cause vitamin D deficiency. Nevertheless, meta‐analyses of intervention studies have demonstrated improvements in depressive symptoms with vitamin D supplementation, especially if subjects are vitamin D deficient or clinically depressed at baseline (Table S3), which would motivate vitamin D replacement in clinical practice.

Depression and cognitive impairment are also common consequences after TBI, with the prevalence of depression ranging from 6 to 77%, and cognitive problems with memory, attention and executive functioning are frequently seen.^9^, ^10^ Vitamin D status may therefore play a role in the development or exacerbation of cognitive and psychiatric problems after TBI, impacting recovery and quality of life. Vitamin D receptors and the vitamin D‐activating enzyme, 1‐alpha‐hydroxylase, are widely distributed in the human brain.^11^ Importantly, animal studies show that vitamin D status can influence behavioural recovery, including memory, and neuropathology after TBI.^4^, ^12^ Neuroinflammation is a common consequence of TBI that may impair recovery,^13^ and may be a linking mechanism for the beneficial effects of vitamin D in rat models of TBI.^4^, ^12^, ^14^

Vitamin D status is best assessed using serum 25‐hydroxy‐cholecalciferol, 25(OH)D3, concentration, as this is the major circulating form of vitamin D with a long half‐life of 2–3 weeks.^15^ United Kingdom national surveys suggest that approximately 20% of adults can have low vitamin D status,^16^ with seasonal variation meaning that in winter this can reach 50% of White adults.^17^ Levels are also lower in subjects with darker skin as melanin absorbs UV‐B radiation from sunlight.^18^ Vitamin D status can be classified as replete, insufficient or deficient, using established criteria (Table S1).

However, there are no published clinical data on the prevalence of vitamin D deficiency in patients after TBI or its association with poorer clinical outcomes. Therefore, the purpose of this retrospective cohort study was to determine the prevalence of vitamin D deficiency and insufficiency after TBI, and whether it is associated with severity of TBI, greater cognitive impairment, worse symptoms and quality of life.

## Methods

### Study design

Case records were retrospectively reviewed from 581 new patients attending the multidisciplinary adult TBI clinic at Charing Cross/St Mary's Hospitals, Imperial College Healthcare NHS Trust (ICHNT), London, UK, between July 2009 and March 2015, to collate data from their first outpatient appointment when vitamin D status was assessed. All data were collected as part of routine clinical care. Patients taking part in neuroimaging research also gave written consent to allow records to be accessed (Research Ethics Committee West London 09/H0707/82).

### Inclusion/exclusion criteria

Inclusion criteria were as follows: (i) either gender, (ii) any age, (iii) experienced at least one TBI of any severity^19^ and (iv) any ethnicity. Exclusion criteria were as follows: (i) no serum vitamin D concentration available; (ii) disorder affecting vitamin D, PTH or calcium physiology including inflammatory bowel disease, severe liver failure, small bowel injury, giardia, chronic diarrhoea, bariatric surgery, gastrostomy feeding, chronic renal failure, primary hyperparathyroidism, on phenytoin; (iii) pre‐TBI disorder affecting quality of life and mood, including psychiatric conditions (attention‐deficit hyperactivity disorder, anxiety, depression, schizophrenia, psychosis, learning difficulty), (iv) diabetes mellitus, current alcohol excess or drug abuse; (v) on vitamin D supplements; (vi) time since TBI >60 months to avoid referral biases leading to worse recovery; and (vii) no ethnicity data.

### Data collection

The following data were collected: (i) patient demographics: age, gender, ethnicity, body mass index (BMI), time since TBI, time of year; (ii) measures of TBI severity: symptomatic, mild, moderate–severe from Mayo classification,^19^ duration of post‐traumatic amnesia (PTA), neurosurgery, epilepsy; (iii) cognitive function measured by Addenbrooke's Cognitive Examination‐Revised (ACE‐R)^20^; (iv) symptom questionnaire scores: Beck Depression Inventory‐II (BDI‐II),^21^ Epworth daytime sleepiness score,^22^ Pittsburgh Sleep Quality Index (PSQI)^23^; (v) quality‐of‐life questionnaire scores: Nottingham Health Profile (NHP),^24^ Short Form‐36 (SF‐36)^25^; and (vi) medical and medication history.

Outcome measures taken >1 month apart from vitamin D measurement were excluded from the analysis due to the potential for recovery over time (>2 months for BMI given its greater stability). BMI measurements from patients with limb amputations were excluded as an inaccurate measure of body composition. As ACE‐R score in part depends on understanding, language and fluency, scores taken from individuals with clear English language difficulties reported in clinic were also excluded (*n* = 12 total, *n* = 6 darker skinned).

### Serum vitamin D and parathyroid hormone

Serum 25(OH)D3 and PTH concentrations were measured by the Department of Chemical Pathology, ICHNT, using Architect chemiluminescent microparticle immunoassay (CMIA) 25(OH)D3 assay and *in vitro* CMIA Architect Intact PTH assay (Abbott Laboratories, Wiesbaden, Germany). To convert vitamin D concentrations from nmol/L to ng/mL divide by 2·496. Vitamin D status was stratified into categories based on the ICHNT guidelines: normal >70 nmol/l (>28·0 ng/ml), insufficient 40–70 nmol/l (16·0–28·0 ng/ml) and deficient <40 nmol/l (<16·0 ng/ml). PTH was stratified into two categories based on standard laboratory ranges: normal 1·1–6·8 pmol/l; high >6·8 pmol/l.

### Covariates

Skin colour was characterized by ethnicity, as either lighter skinned (including White, European Caucasian, Middle Eastern, Chinese or South East Asian) or darker skinned (Black, Indian subcontinent, South Asian, or Mixed including these ethnicities). Season was determined using the date of vitamin D sampling. Winter referred to samples taken during Daylight Saving Time (DST) (1st November to 31st March), and summer to British Summer Time (BST) (1st April to 31st October).

### Statistical analysis

Data were analysed using SPSS v.22 (IBM, Armonk, NY, USA). Each variable was tested for normality using the Kolmogorov–Smirnov test. Data are presented as median (interquartile range) (min–max) as they were non‐normally distributed. Nonparametric Kruskal–Wallis one‐way anova was used to determine differences between the three vitamin D status groups for continuous variables, and Mann–Whitney *U*‐test for the two PTH category groups. Chi‐squared tests were performed to determine differences between groups in categorical demographic variables.

Two‐way anova with *post hoc* least significance difference (LSD) test determined the influence of skin colour and season between‐subject variables on serum vitamin D concentrations. One‐way ancova with *post hoc* LSD test determined differences between vitamin D groups when controlling for covariates: age, gender, time since TBI and severity of TBI (classified as symptomatic mild or moderate–severe, or as PTA less or greater than 1 week). Log_10_‐transformed values were used for age and time since TBI, as they were not normally distributed.

Spearman's bivariate correlation coefficient examined the relationship between vitamin D concentrations and outcome measures. Partial correlation coefficients were determined when including covariates: age, gender, time since TBI and severity of TBI, in the model.

Significance was taken as *P* < 0·05. Due to multiple comparisons between the subdomains of quality‐of‐life questionnaires, Bonferroni correction was applied to SF‐36 and NHP scores, with significance taken as *P* < 0·006 in SF‐36 (9 subdomains) and *P* < 0·008 in NHP (6 subdomains).

## Results

### Cohort demographics

**Figure 1:**
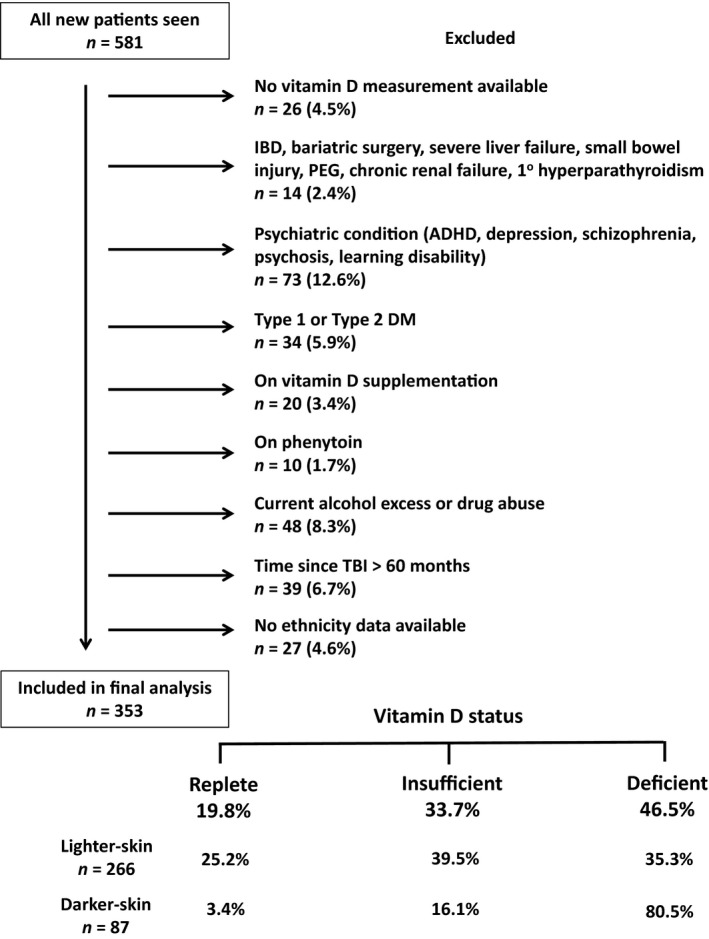
Patient inclusion and exclusion flow chart. Data on prevalence of vitamin D status are given at bottom of chart. Abbreviations: ADHD, attention‐deficit hyperactivity disorder; DM, diabetes mellitus; IBD, inflammatory bowel disease; PEG, percutaneous endoscopic gastrostomy; TBI, traumatic brain injury.

After exclusion criteria were applied, 353 patients were included in the final analysis (Fig. 1). Only 3% of patients were excluded as they were on vitamin D supplementation. Demographic characteristics of the cohort are shown in Table 1 and Table S4. As typical for TBI, patients were generally young men (median age 35·1 years, 74·8% male). 75·4% were of the lighter skinned ethnic categories. The causes of TBI were as follows: road traffic accidents (33%), falls (31%), assault (17%), sports injuries (6%), improvised explosive device blast TBI (5%), object falling on head (5%), other (1%) and unknown (2%).

**Table 1:** Demographic characteristics of cohort

Variable	Units	*n*	All subjects	*n*	Vitamin D replete	*n*	Vitamin D insufficient	*n*	Vitamin D deficient	H or χ statistic	*P*
Age	(years)	353	35·1 [26·6,48·3] (16·6–88·1)	70	37·9 [26·8,53·4] (17·9–86·7)	119	36·4 [28·3,50·2] (19·9–87·1)	164	32·8 [24·6,46·6]* (16·6–88·1)	6·1	**0·047**
Male	*n* (%)	353	264 (74·8%)	70	47 (67·1%)	119	86 (72·3%)	164	131 (79·9%)	4·8	0·090^†^
Darker‐skinned	*n* (%)	353	87 (24·6%)	70	3 (4·3%)	119	14 (11·8%)	164	70 (42·7%)*, ^#^	55·0	**<0·001** ^†^
BMI	(kg/m^2^)	325	25·0 [22·4,27·8] (14·8–44·0)	67	24·3 [21·6,27·3] (14·8–44·0)	113	26·2 [23·5,29·1]** (14·8–44·0)	145	24·8 [22·2,27·5]^##^ (15·6–37·2)	10·4	**0·005**
Time since TBI	(months)	353	4·4 [2·6,9·8] (0·3–56·5)	70	4·4 [2·8,10·8] (1·0–35·4)	119	5·0 [2·8,10·4] (0·7–53·0)	164	3·5 [2·3,9·6) (0·3–56·5)	5·2	0·075
Winter	*n* (%)	353	161 (45·6%)	70	16 (22·9%)	119	46 (38·7%)*	164	99 (60·4%)*, ^#^	31·3	**<0·001** ^†^
Vitamin D	(nmol/l)	353	42·0 [28,6,63·9] (10·0–163·0)	70	82·5 [75·0, 96.7] (70·0–163·0)	119	51·7 [46·0,59·8]*** (40·0–69·2)	164	27·7 [21·1,34·3]***, ^##^, ^#^ (10·0–39·9)	300·5	**<0·001**
PTH	(pmol/l)	337	5·0 [3·4,6·5] (1·3–17·7)	70	4·2 (3·0,5·3] (1·8–12·4)	113	4·5 (3·2,6·2)** (1·4–14·0)	154	5·4 [4·3,7·2]*** (1·3–17·7)	21·1	**<0·001**
High PTH	(pmol/l)	337	72 (20·4%)	70	8 (11·4%)	113	20 (16·8%)	154	44 (26·8%)*, ^#^	9·8	**0·008** ^†^

Values stated as median [interquartile range] (min‐max) or *n* (%) for categorical variables.

Statistical results from Kruskal‐Wallis one‐way anova test, except for ^†^Chi‐squared test where *post‐hoc* results only displayed as *P* < 0·05. Post‐hoc comparison between groups: *P < 0·05, **P < 0·01, ***P < 0·001 *vs* vitamin D replete; ^#^ *P* < 0·05, ^##^ *P* < 0·01, ^###^ *P* < 0·001 *vs* vitamin D insufficient. . *P* values in bold text indicate statistically significant results for ANOVA. Abbreviations: BMI, body mass index; PTH, parathyroid hormone; TBI, traumatic brain injury. To convert vitamin D concentrations from nmol/l to ng/ml divide by 2·496.

### Vitamin D status

In total, 46·5% of patients had vitamin D deficiency and an additional 33·7% were insufficient, with only 19·8% being replete (Fig. 1). The expected relationship between low vitamin D and high PTH concentrations was seen (Table 1, Figure S1). There was a significant difference in age, skin colour, BMI, and season between the three vitamin D categories, but no differences were found in gender or time since TBI (Table 1). Patients with vitamin D deficiency were significantly younger than those who were replete (*P* < 0·05). There were a greater proportion of darker skinned ethnicities present in the vitamin D‐deficient group compared with the other two groups (*P* < 0·05). Patients with vitamin D insufficiency also had a higher BMI compared with patients in the other two groups. A significantly higher proportion of samples were taken in the winter in patients with vitamin D deficiency.

### Season and skin colour

**Figure 2:**
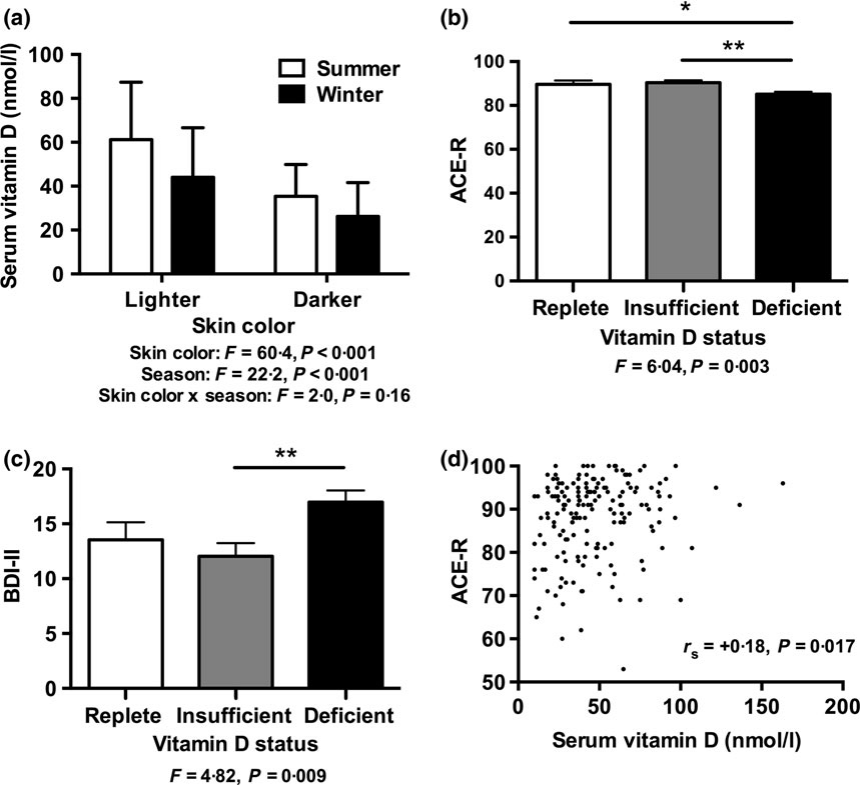
Relationship between Vitamin D status and skin colour, season, cognition and depressive symptoms. (a) Influence of skin colour and season on serum vitamin D concentrations; (b, c) influence of vitamin D status on (b) cognition assessed by ACE‐R scores and (c) depressive symptoms assessed by BDI‐II; and (d) correlation between serum vitamin D concentrations and ACE‐R score. Statistical results from (a) two‐way anova including skin colour and ethnicity as between‐subject factors, indicating a significant effect of skin colour and season but no interaction effect (*n* = 353); (b, c) one‐way ancova including vitamin D status group as between‐subject factor, correcting for age, gender, time since TBI and PTA duration >1 week, *P < 0·05, **P < 0·01 (b: *n* = 27 replete, *n* = 66 insufficient, *n* = 77 deficient; c: *n* = 53 replete, *n* = 95 insufficient, *n* = 123 deficient); (d) *r*_s_ indicates Spearman's correlation coefficient, *n* = 175. Abbreviations: ACE‐R, Addenbrooke's Cognitive Examination‐Revised, BDI‐II, Beck Depression Inventory‐II; PTA, post‐traumatic amnesia; TBI, traumatic brain injury. To convert vitamin D concentrations from nmol/l to ng/ml divide by 2·496.

Vitamin D concentrations were affected by both season and skin colour (Fig. 2a). Darker skin ethnicities had significantly lower vitamin D compared with lighter skin ethnicities (effect size mean ± SEM (95% CI) 21·8 ± 2·8 nmol/l (16·3, 27·3), *F*(1,349) = 60·4, *P* < 0·001, Cohen's d = 0·83). Vitamin D concentrations were significantly lower in the winter compared with the summer (effect size 13·2 ± 2·8 (7·7, 18·7), *F*(1,349) = 22·2, *P* < 0·001, Cohen's d = 0·34). There was, however, no interaction between skin colour and season on vitamin D (*F*(1,349) = 1·970, *P* = 0·16).

### Comparison with general population

To gain an estimation of whether the prevalence of vitamin D insufficiency/deficiency after TBI was greater than would be expected in the general population, our results were compared with a UK nationwide cohort study of White British subjects aged 45 years (Table 2).^17^ The prevalence of vitamin D concentrations <40 and <75 nmol/l in White British and European Caucasian patients after TBI was significantly higher than in the general population in summer (22 *vs* 15%, *P* = 0·037; 70 *vs* 61%, *P* = 0·022), but not in winter.

**Table 2:** Prevalence of vitamin D deficiency in white Caucasians compared to a UK survey

	*n*	Vitamin D		
		<25 nmol/l	<40 nmol/l	<75 nmol/l
DST/Winter				
Current Audit	**119**	**21 (17·6%)**	**63 (52·9%)**	**108 (90·8%)**
UK Survey	2850	441 (15·5%)	1328 (46·6%)	2482 (87·1%)
Chi Statistic		0·41	1·85	2·76
*P* Value		0·52	0·17	0·097
BST/Summer				
Current Audit	**142**	**5 (3·5%)**	**31 (21·8%)**	**100 (70·4%)**
UK Survey	4587	147 (3·2%)	706 (15·4%)	2793 (60·9%)
Chi Statistic		0·044	4·34	5·27
*P* Value		0·83	**0·037**	**0·022**

Prevalence of vitamin D status in current audit (for white Caucasians only) was compared using chi‐squared test to UK survey data for white Caucasians reported by Hypponen E and Power C. Hypovitaminosis D in British adults at age 45 year: nationwide cohort study of dietary and lifestyle predictors. Am J Clin Nutr 2007 85:860–868.

Abbreviations: BST, British Summer Time; DST, Daylight Saving Time; UK, United Kingdom.

To convert vitamin D concentrations from nmol/l to ng/ml divide by 2·496. *P* values in bold text indicate statistically significant results.

### Markers of TBI severity

There was no significant difference in markers of TBI severity: PTA duration, or prevalence of moderate–severe TBI, craniotomy or epilepsy between the vitamin D groups (Table 3). This remained nonsignificant even after correction for age, skin colour and season.

**Table 3:** Relationship between vitamin D status and markers of TBI severity

Variable	Units	*n*	All subjects	*n*	Vitamin Dreplete	*n*	Vitamin D insufficient	*n*	Vitamin D deficient	Uncorrected		Corrected for: age, skin color season	
										χ statistic	*P*	*F* statistic	*P* *
Moderate‐Severe TBI	*n* (%)	353	263 (74·5%)	70	51 (72·9%)	119	93 (78·2%)	164	119 (72·6%)	1·26	0·53	0·99 F(2,347)	0·59
PTA > 1 day	*n* (%)	328	154 (43·3%)	66	33 (47·1%)	109	55 (46·2%)	153	66 (40·2%)	1·68	0·43	1·06 F(2,321)	0·35
PTA > 1 week	*n* (%)	328	67 (19·0%)	66	13 (18·6%)	109	27 (22·7%)	153	27 (16·5%)	2·01	0·37	1·13 F(2,321)	0·33
Craniotomy	*n* (%)	353	2 (0·6%)	70	1 (1·4%)	119	1 (0·8%)	164	0 (0%)	2·02	0·37	1·99 F(2,347)	0·14
Epilepsy	*n* (%)	353	47 (13·3%)	70	10 (14·3%)	119	16 (13·4%)	164	21 (12·8%)	0·10	0·95	0·41 F(2,347)	0·67

Values stated as *n* (%).

Statistical results from Chi‐squared test, except for *one way ancova when controlling for covariates. Abbreviations: PTA, post‐traumatic amnesia; TBI, traumatic brain injury.

### Vitamin D status and outcome measures

Patients with Vitamin D deficiency had significantly lower ACE‐R scores compared to patients with vitamin D insufficiency (*P* = 0·003) (Table 4). This significance remained after correction for age, gender, time since TBI and TBI severity, using either PTA >1 week (effect size 5·2 ± 1·6 (95% CI 2·1–8·3), *P* = 0·001, Cohen's d = 0·56) (Fig. 2b), or prevalence of moderate–severe TBI (effect size 4·9 ± 1·5 (1·9–7·9), *P* = 0·002, Cohen's d = 0·54), as markers of severity. After correction for these factors, patients with vitamin D deficiency also had significantly lower ACE‐R scores than patients who were replete (effect size 4·5 ± 2·1 (0·3–8·6), *P* = 0·034, Cohen's d = 0·48).

**Table 4:** Relationship between vitamin D status and cognition, symptoms and quality of life

Questionnaire	Max score	*n*	All Subjects	Vitamin D replete	Vitamin D insufficient	Vitamin D deficient	Uncorrected		Corrected for: age, gender, moderate‐severe TBI, time since TBI		Corrected for: age, gender, PTA>1 week, time since TBI		
							H statistic	*P*	*F* statistic	*P* *	*n*	*F* statistic	*P* *
ACE‐R	100	175	91 [82,95] (53–100)	91 [85,95] (69–100)	93 [87,96] (53–100)	88 [79,93]^##^(60–100)	9·22	**0·010**	3·03 *F*(2,168)	**0·005**	170	6·04 F(2,163)	**0·003**
BDI‐II	63	285	12 [6,21] (0–60)	13 [4,24] (0–37)	11 [5,19] (0–38)	12 [6,24] (0–60)	3·05	0·22	4·15 *F*(2,278)	**0·017**	271	4·82 F(2,264)	**0·009**
Epworth	24	260	7 [2,11] (0–22)	6 [2,10] (0–17)	8 [3,12] 10–22)	7 [3,11] (0–20)	2·42	0·30	1·46 *F*(2,253)	0·23	247	0·97 F(2,240)	0·38
PSQI	21	134	7 [5,11] (0–19)	7 [6,11] (1–18)	6 [4,10] (1–19)	7 [4,12] (0–19)	1·27	0·53	0·59 *F*(2,127)	0·55	125	0·80 F(2,118)	0·45
SF‐36 (QoL)^†^													
Physical Functioning	100	177	70 [48,91] (0–100)	63 [21,90] (5–100)	75 [55,95] (20–100)	70 [46,92] (0–100)	3·93	0·14	2·59 *F*(2,170)	0·078	166	3·16 F(2,159)	0·045
Role‐Physical	100	168	0 [0,75] (0–100)	0 [0,75] (0–100)	0 [0,94] (0–100)	0 [0,75] (0–100)	0·37	0·83	0·18 *F*(2,161)	0·83	159	0·26 F(2,152)	0·77
Role‐Emotional	100	167	33 [0,100] (0–100)	0 [0,67] (0–100)	33 [0,100] (0–100)	67 [0,100] (0–100)	5·05	0·080	1·69 *F*(2,160)	0·19	158	1·74 F(2,151)	0·18
Energy Fatigue	100	175	45 [25,60] (0–95)	43 [25,60] (5–90)	43 [25,56] (0–95)	45 [28,60] (0–95)	0·20	0·91	0·02 *F*(2,168)	0·98	165	0·00 F(2,158)	0·99
Emotional Wellbeing	100	176	60 [48,80] (0–100)	56 [41,71] (12–92)	62 [48,80] (0–96)	64 [48,80] (0–100)	2·52	0·28	0·64 *F*(2,169)	0·53	166	0·33 F(2,159)	0·72
Social Functioning	100	178	50 [25,75] (0–100)	50 [25,75] (13–100)	50 [25,75] (0–100)	50 [25,75] (0–100)	0·58	0·75	0·25 *F*(2,171)	0·78	168	0·27 F(2,161)	0·76
Pain	100	178	58 [33,80] (0–100)	45 [33,90] (0–100)	58 [39,90] (0–100)	59 [34,78] (0–100)	0·43	0·81	0·26 *F*(2,171)	0·78	168	0·34 F(2,161)	0·72
General Health	100	179	55 [40,75] (0–100)	60 [33,75] (0–100)	53 [40,71] (5–100)	55 [35,75] (0–100)	0·30	0·86	0·16 *F*(2,172)	0·86	168	0·30 F(2,151)	0·74
Health Change	100	176	25 [25,50] (0–100)	25 [0,50] (0–100)	25 [25,50] (0–100)	25 [25,50] (0–100)	4·39	0·11	2·38 *F*(2,169)	0·095	165	2·27 F(2,158)	0·11
NHP (QoL)^‡^													
Energy Levels	100	172	39 [0,100] (0–100)	62 [6,100] (0–100)	37 [0,63] (0–100)	37 [0,100] (0–100)	1·95	0·38	0·77 *F*(2,165)	0·47	161	1·44 F(2,154)	0·24
Pain	100	166	9 [0,33] (0–100)	13 [0,38] (0–100)	0 [0,18] (0–100)	8 [0,36] (0–100)	1·37	0·51	0·94 *F*(2,159)	0·40	156	1·87 F(2,149)	0·16
Emotional Reaction	100	161	20 [0,53] (0–100)	30 [10,63] (0–90)	17 [0,40] (0–93)	23 [0,62] (0–100)	3·01	0·22	1·68 *F*(2,154)	0·19	151	2·25 F(2,144)	0·11
Sleep	100	164	28 [0,57] (0–100)	34 [10,52] (0–100)	15 [0,50] (0–100)	79 [0,61] (0–100)	1·22	0·54	0·59 *F*(2,157)	0·55	154	0·55 F(2,147)	0·58
Social Isolation	100	164	0 [0,54] (0–100)	23 [0,62] (0–100)	0 [0,37] (0–100)	0 [0,64] (0–100)	4·57	0·10	2·06 *F*(2,157)	0·13	154	2.29 F(2,147)	0·11
Physical Activity	100	163	9 [0,31] (0–100)	11 [0,34] (0–79)	0 [0,21] (0–67)	0 [0,33] (0–100)	1·55	0·46	1·24 *F*(2,156)	0·29	153	1·68 F(2,146)	0·19
Average	100	152	22 [7,43] (0–98)	26 [17,50] (0–81)	19 [6,34] (0–72)	23 [7,48] (0–98)	3·10	0·21	1·93 *F*(2,145)	0·15	142	2·72 F(2,135)	0·069
NHP‐II	7	176	3 [1,6] (0–7)	4 [2,6] (0–7)	3 [1,5] (0–7)	3 [1,6] (0–7)	2·63	0·27	1·54 *F*(2,169)	0·22	166	2·09 F(2,159)	0·13

Values stated as median [interquartile range] (min‐max)

Statistical results from Kruskal‐Wallis one‐way anova test, except for *one way ancova when controlling for covariates

^##^ *P* < 0·01 *vs* vitamin D insufficient. *P* values in bold text indicate statistically significant results for ANOVA.

Abbreviations: ACE‐R, Addenbrooke's Cognitive Examination Revised; BDI‐II, Beck Depression Inventory II; NHP, Nottingham Health Profile; PSQI, Pittsburgh

Sleep Quality Index; QoL, quality of life; SF‐36, Short Form 36 Health Survey.

^†^SF‐36: higher score = better performance.

^‡^NHP: lower score = better performance.

In stepwise linear regression, vitamin D status (F‐to‐enter = 6·70, *r* = +0·18, *P* = 0·010), but not skin colour (*P* = 0·57) or season (*F* = 0·28), also correlated with ACE‐R score. Additionally, this significance also remained when the analysis was restricted to just lighter skin ethnic individuals, in order to exclude many of those who may be less proficient in English as a second language (*F*(2,124) = 4·5, *P* = 0·013; effect size *vs* insufficient, 5·4 ± 1·8 (1·8–9·1), *P* = 0·004, Cohen's d = 0·59; effect size *vs* replete, 4·6 ± 2·3 (0·01–9·11), *P* = 0·050, Cohen's d = 0·49).

In the uncorrected model, vitamin D status was not significantly associated with BDI‐II score (Table 4). However, patients with vitamin D deficiency had significantly higher BDI‐II scores compared to those with insufficiency when corrected for age, gender, time since TBI and TBI severity, using both PTA >1 week (effect size 4·9 ± 1·6 (1·7–8·1), *P* = 0·003, Cohen's d = 0·42) (Fig. 2c) or prevalence of moderate–severe TBI (effect size 4·3 ± 1·5 (1·3–7·4), *P* = 0·005, Cohen's d = 0·38). Season had no significant effect on BDI‐II score (winter vs. summer effect size −1·5 ± 1·3 (−4·2, 1·1), *P* = 0·25, Cohen's d = 0·14).

Vitamin D status did not influence Epworth daytime sleepiness or PSQI scores, or any components of the SF‐36 and NHP questionnaires, even after correction for age, gender, time since TBI and TBI severity (Table 4).

### Vitamin D concentrations and outcome measures

Vitamin D concentrations were significantly correlated with performance on the ACE‐R (*r*_s_ = +0·18, *P* = 0·017) (Table S5). This remained significant when corrected for age, gender, time since TBI and TBI severity, using both PTA >1 week (*r*_s_ = +0·18, *P* = 0·021) (Fig. 2d) and prevalence of moderate–severe TBI (*r*_s_ = +0·18, *P* = 0·017).

There was, however, no significant correlation between vitamin D concentrations and symptom scores on BDI‐II, Epworth or PSQI, or quality‐of‐life measures from SF‐36 or NHP (Table S5).

### PTH status and outcome measures

There was no significant association between PTH status, defined as high or normal, and cognition, symptoms scores or quality‐of‐life measures in either the whole cohort (*P* = 0·21–0·94), or those that were vitamin D deficient (*P* = 0·14–0·99), insufficient (*P* = 0·07–0·96) or nonreplete (*P* = 0·10–0·93).

## Discussion

This study has found that vitamin D deficiency is common in patients after TBI with 46·5% of patients being vitamin D deficient, and a further 33·7% being insufficient, with overall 80·2% having low concentrations. This prevalence of vitamin D insufficiency/deficiency in summer in White and European Caucasian individuals after TBI was greater than expected in the general population (using a UK nationwide cohort study of the White British population aged 45 years).^17^ This was despite the fact that the UK survey included areas of greater latitude, such as Scotland, with higher prevalence of vitamin D deficiency, which would be expected to attenuate this difference.

In agreement with other studies in non‐TBI patients,^7^, ^8^ this study found that low vitamin D status was negatively correlated with cognition as assessed by the ACE‐R, even after adjustment for factors that may influence performance, including age, gender, time since TBI and TBI severity.^26^, ^27^, ^28^

When corrected for age, gender, and other factors that may influence BDI‐II score,^29^, ^30^ vitamin D deficiency was also associated with more severe depressive symptoms. This is in agreement with previous meta‐analyses in non‐TBI patients, which show an association between vitamin D concentrations and depression (Table S2).^5^, ^6^

These associations with cognition and depressive symptoms may result from a role for vitamin D in brain function.^31^ Many brain cell types and regions can synthesize the active form of vitamin D, 1,25(OH)D3, including the hypothalamus and substantia nigra,^11^ areas implicated in the pathophysiology of depression. Vitamin D modulates the production of neuroprotective factors and neurotransmitters (including growth factors and choline acetyltransferase), neuronal apoptosis, neuroinflammation, oxidative stress, excitotoxicity, and myelin and axon repair.^8^, ^32^

In rat models of TBI, vitamin D deficiency leads to greater open‐field behavioural deficits and attenuates the beneficial effects of progesterone administration.^4^ This is reversed by co‐administration of vitamin D,^4^ which also improves spatial memory processing.^12^ Changes in neuroinflammation may be a linking mechanism as vitamin D deficiency is associated with elevated brain inflammatory proteins with, and even without, TBI.^4^ Vitamin D administration reduces neuronal loss and modifies the proliferation of reactive astrocytes.^12^, ^14^

There was no association found between vitamin D status and sleep, or any quality‐of‐life domains on the NHP and SF‐36. This is contradictory to previous research in premenopausal women, and women with chronic pain (unlike current study where 74·8% were male), where vitamin D deficiency is associated with worse SF‐36 and NHP quality‐of‐life scores, particularly in vitality, physical and mental component subscores.^33^, ^34^ However, there are many factors that may affect quality of life, in addition to mood and cognitive function.

It is hypothesized that TBI may increase the prevalence of vitamin D insufficiency/deficiency as a result of hospitalization, impaired social functioning and absence from work, reducing sun exposure. However, in this audit, no markers of TBI severity influenced vitamin D status. This may be related to type II errors as some of the measures, such as need for neurosurgery and presence of epilepsy, were uncommon. In addition, TBI severity determined by duration of PTA and presence of intracranial pathology may not be relevant as a risk factor for sun exposure, given that the median time after TBI was 4·4 months.

Furthermore, vitamin D plays a major role in bone homoeostasis and can reduce risk of fracture and rate of bone loss in those over 50 years old.^35^ Thus, irrespective of the relationship between vitamin D status and cognition/depression, screening and treating patients after TBI could be justified to improve healing of any associated fractures, and bone health, especially as they become less active. This is a neglected treatment avenue as only 3% of our cohort were taking vitamin D supplements when vitamin D status was initially assessed at their first TBI clinic appointment.

There are several limitations with this study. Most importantly, this is an association study, and so the direction of causality seen in the results is unknown. Although vitamin D deficiency may be contributing to impaired cognitive function or higher depressive symptoms, this cannot be proven from this study. TBI itself can cause these symptoms, leading to individuals spending more time indoors and thus becoming more vitamin D deficient. However, meta‐analyses of intervention studies in non‐TBI patients have shown that vitamin D supplementation improves depressive symptoms, suggesting causation (Table S3). There is a currently a lack of intervention studies looking at the effects of vitamin supplementation and cognition, and further work is needed to clarify the direction of this association. Furthermore, this audit did not include a control group to allow comparison of prevalence data in individuals utilizing the same vitamin D assay. Finding a suitable control group from other studies is difficult due to differences in demographics and variability in the assay used.

There are also limitations regarding categorizing skin colour based on ethnicities of patients. Although Middle Eastern and eastern European individuals were considered to be lighter skinned, they may have greater levels of melanin than White Caucasians. Similarly, the categorization of season into DST and BST does not allow separation of all four seasons. However, appropriate relationships were seen between skin colour and season with vitamin D in our study, indicating that our approach gave biologically relevant results.^17^, ^18^ Reduced strength and duration of sunlight during winter and diversity of ethnicity in the UK will increase the clinical risk of vitamin D deficiency in our TBI clinic population.

Due to the potential limitations with the ACE‐R in those less proficient in English, a further development would be to use only measures of cognitive function that do not depend on language, such as memory, visuospatial and attention/orientation subcomponents. Alternatively, neuropsychological tests more specific to consequences after TBI could be used, for example focusing on planning, concentration and switching.

In conclusion, vitamin D deficiency is common in patients after TBI and is associated with worse cognitive function and more severe depressive symptoms. There is a potential for vitamin D to be a modifiable risk factor to improve recovery after TBI. Further work is needed to examine the longitudinal effects of vitamin D status and supplementation on neurocognitive and psychological function and neuroimaging biomarkers to determine whether assessment and treatment of vitamin D deficiency should become part of clinical guidelines in the management of TBI.

## Author's contributions

OAJ and APG designed the study; OAJ, CF, JZ, AM, MEKN, CT, TEH, SRJ, PJ, GS, LL, NG, DB, DJS and APG helped in data collection; OAJ and APG contributed to data analysis, data interpretation and writing of manuscript; all authors reviewed and approved the manuscript. APG is the guarantor of this work and, as such, had full access to all the data in the study and takes responsibility for the integrity of the data and the accuracy of the data analysis.

## Disclosure

The authors have nothing to declare.

## Supporting information


**Figure S1.** Relationship between serum vitamin D and PTH concentrations.
**Table S1.** Guidelines for definition of vitamin D status
**Table S2**. Summary of meta‐analyses of association studies between vitamin D status and depression
**Table S3**. Summary of meta‐analyses of intervention studies with vitamin D supplementation and depression.
**Table S4**. Demographics in outcome variable sub‐groups.
**Table S5**. Correlations between vitamin D concentration and cognition, symptoms and quality of life

## References

[1] Nice . (2014) National Institute For Health And Care Excellence Guidance. Head Injury: Assessment And Early Management. Http://Www.Nice.Org.Uk/Guidance/Cg176,

[2] Tanriverdi, F. & Kelestimur, F. (2015) Pituitary dysfunction following traumatic brain injury: clinical perspectives. Neuropsychiatric Disease and Treatment, 11, 1835–1843. 26251600 10.2147/NDT.S65814 PMC4524578

[3] Rajakumar, K. , Fernstrom, J.D. , Holick, M.F. *et al* (2008) Vitamin D status and response to vitamin D3 in obese vs. non‐obese African American Children. Obesity, 16, 90–95. 18223618 10.1038/oby.2007.23

[4] Cekic, M. , Cutler, S.M. , Vanlandingham, J.W. *et al* (2011) Vitamin D deficiency reduces the benefits of progesterone treatment after brain injury in aged rats. Neurobiology of Aging, 32, 864–874. 19482377 10.1016/j.neurobiolaging.2009.04.017 PMC3586224

[5] Ju, S.Y. , Lee, Y.J. & Jeong, S.N. (2013) Serum 25‐hydroxyvitamin D levels and the risk of depression: a systematic review and meta‐analysis. The Journal of Nutrition, Health & Aging, 17, 447–455. 10.1007/s12603-012-0418-0 PMC12878238 23636546

[6] Anglin, R.E. , Samaan, Z. , Walter, S.D. *et al* (2013) Vitamin D deficiency and depression in adults: systematic review and meta‐analysis. British Journal of Psychiatry, 202, 100–107. 23377209 10.1192/bjp.bp.111.106666

[7] Etgen, T. , Sander, D. , Bickel, H. , *et al* (2012) Vitamin D deficiency, cognitive impairment and dementia: a systematic review and meta‐analysis. Dementia and Geriatric Cognitive Disorders, 33, 297–305. 22759681 10.1159/000339702

[8] Balion, C. , Griffith, L.E. , Strifler, L. *et al* (2012) Vitamin D, cognition, and dementia: a systematic review and meta‐analysis. Neurology, 79, 1397–1405. 23008220 10.1212/WNL.0b013e31826c197f PMC3448747

[9] Kreutzer, J.S. , Seel, R.T. & Gourley, E. (2001) The prevalence and symptom rates of depression after traumatic brain injury: a comprehensive examination. Brain Injury, 15, 563–576. 11429086 10.1080/02699050010009108

[10] Arciniegas, D.B. , Held, K. & Wagner, P. (2002) Cognitive impairment following traumatic brain injury. Current Treatment Options in Neurology, 4, 43–57. 11734103 10.1007/s11940-002-0004-6

[11] Eyles, D.W. , Smith, S. , Kinobe, R. *et al* (2005) Distribution of the vitamin D receptor and 1α‐hydroxylase in human brain. Journal of Chemical Neuroanatomy, 29, 21–30. 15589699 10.1016/j.jchemneu.2004.08.006

[12] Hua, F. , Reiss, J.I. , Tang, H. *et al* (2012) Progesterone and low‐dose vitamin d hormone treatment enhances sparing of memory following traumatic brain injury. Hormones and Behavior, 61, 642–651. 22570859 10.1016/j.yhbeh.2012.02.017 PMC3517217

[13] Hinson, H.E. , Rowell, S. & Schreiber, M. (2015) Clinical evidence of inflammation driving secondary brain injury: a systematic review. The Journal of Trauma and Acute Care Surgery, 78, 184–191. 25539220 10.1097/TA.0000000000000468 PMC4297199

[14] Tang, H. , Hua, F. , Wang, J. *et al* (2013) Progesterone and vitamin D: improvement after traumatic brain injury in middle‐aged rats. Hormones and Behavior, 64, 527–538. 23896206 10.1016/j.yhbeh.2013.06.009 PMC3833454

[15] Hollis, B.W. (1996) Assessment of vitamin D nutritional and hormonal status: what to measure and how to do it. Calcified Tissue International, 58, 4–5. 8825231 10.1007/BF02509538

[16] Nice . (2014) National Institute For Health And Care Excellence Guidance. Vitamin D: Increasing Supplement Use In At‐Risk Groups. Http://Www.Nice.Org.Uk/Guidance/Ph56,

[17] Hypponen, E. & Power, C. (2007) Hypovitaminosis D in British adults at age 45 y: nationwide cohort study of dietary and lifestyle predictors. American Journal of Clinical Nutrition, 85, 860–868. 17344510 10.1093/ajcn/85.3.860

[18] Harris, S.S. (2006) Vitamin D and African Americans. Journal of Nutrition, 136, 1126–1129. 16549493 10.1093/jn/136.4.1126

[19] Malec, J.F. , Brown, A.W. , Leibson, C.L. et al. (2007) The Mayo classification system for traumatic brain injury severity. Journal of Neurotrauma, 24, 1417–1424. 17892404 10.1089/neu.2006.0245

[20] Mioshi, E. , Dawson, K. , Mitchell, J. et al. (2006) The Addenbrooke's Cognitive Examination Revised (ACE‐R): a brief cognitive test battery for dementia screening. International Journal of Geriatric Psychiatry, 21, 1078–1085. 16977673 10.1002/gps.1610

[21] Beck, A.T. , Steer, R.A. , Ball, R. et al. (1996) Comparison of Beck Depression Inventories ‐Ia and ‐II in psychiatric outpatients. Journal of Personality Assessment, 67, 588–597. 8991972 10.1207/s15327752jpa6703_13

[22] Johns, M.W. (1991) A new method for measuring daytime sleepiness: the Epworth sleepiness scale. Sleep, 14, 540–545. 1798888 10.1093/sleep/14.6.540

[23] Buysse, D.J. , Reynolds, C.F. , Monk, T.H. et al. (1989) The Pittsburgh Sleep Quality Index: a new instrument for psychiatric practice and research. Psychiatry Research, 28, 193–213. 2748771 10.1016/0165-1781(89)90047-4

[24] Wiklund, I. (1990) The Nottingham Health Profile ‐ a measure of health‐related quality of life. Scandinavian Journal of Primary Health Care. Supplement, 1, 15–18. 2100359

[25] Brazier, J.E. , Harper, R. , Jones, N.M. et al. (1992) Validating the SF‐36 health survey questionnaire: new outcome measure for primary care. BMJ, 305, 160–164. 1285753 10.1136/bmj.305.6846.160 PMC1883187

[26] Kimura, D. (1996) Sex, sexual orientation and sex hormones influence human cognitive function. Current Opinion in Neurobiology, 6, 259–263. 8725969 10.1016/s0959-4388(96)80081-x

[27] Novack, T.A. , Alderson, A.L. , Bush, B.A. et al. (2000) Cognitive and functional recovery at 6 and 12 months post‐TBI. Brain Injury, 14, 987–996. 11104138 10.1080/02699050050191922

[28] Schretlen, D.J. & Shapiro, A.M. (2003) A quantitative review of the effects of traumatic brain injury on cognitive functioning. International Review of Psychiatry, 15, 341–349. 15276955 10.1080/09540260310001606728

[29] Weissman, M.M. & Klerman, G.L. (1985) Gender and depression. Trends in Neurosciences, 8, 416–420.

[30] Meltzer, C.C. , Smith, G. , Dekosky, S.T. et al. (1998) Serotonin in aging, late‐life depression, and Alzheimer's disease: the emerging role of functional imaging. Neuropsychopharmacology, 18, 407–430. 9571651 10.1016/S0893-133X(97)00194-2

[31] Mccann, J.C. & Ames, B.N. (2008) Is there convincing biological or behavioral evidence linking vitamin D deficiency to brain dysfunction? FASEB Journal, 22, 982–1001. 18056830 10.1096/fj.07-9326rev

[32] Groves, N.J. , Mcgrath, J.J. & Burne, T.H. (2014) Vitamin D as a neurosteroid affecting the developing and adult brain. Annual Review of Nutrition, 34, 117–141. 10.1146/annurev-nutr-071813-105557 25033060

[33] Ecemis, G.C. & Atmaca, A. (2013) Quality of life is impaired not only in vitamin D deficient but also in vitamin D‐insufficient pre‐menopausal women. Journal of Endocrinological Investigation, 36, 622–627. 23511484 10.3275/8898

[34] Kuru, P. , Akyuz, G. , Yagci, I. et al. (2015) Hypovitaminosis D in widespread pain: its effect on pain perception, quality of life and nerve conduction studies. Rheumatology International, 35, 315–322. 25085713 10.1007/s00296-014-3099-7

[35] Tang, B.M.P. , Eslick, G.D. , Nowson, C. et al. (2007) Use of calcium or calcium in combination with vitamin D supplementation to prevent fractures and bone loss in people aged 50 years and older: a meta‐analysis. Lancet, 370, 657–666. 17720017 10.1016/S0140-6736(07)61342-7

