# Supplementary material for: Prevalence and correlates of vitamin D deficiency in adults after traumatic brain injury
Source: Clin Endocrinol (Oxf). 2016 Mar 28;85(4):636–44. doi: 10.1111/cen.13045 (PMC5053278; doi:10.1111/cen.13045)
Supplement: Supplementary file 1 — Figure S1. Relationship between serum vitamin D and PTH concentrations. Table S1. Guidelines for definition of vitamin D status Table S2. Summary of meta‐analyses of association studies between vitamin D status and depression Table S3. Summary of meta‐analyses of intervention studies with vitamin D supplementation and depression. Table S4. Demographics in outcome variable sub‐groups. Table S5. Correlations between vitamin D concentration and cognition, symptoms and quality of life [file CEN-85-636-s001.docx]

**SUPPLEMENTARY INFORMATION**

**Prevalence and Correlates of Vitamin D Deficiency in Adults after Traumatic Brain Injury**

Omer A Jamall, Claire Feeney, Joanna Zaw-Linn, Aysha Malik, Mari EK Niemi, Carmen Tenorio-Jimenez, Timothy E Ham, Sagar R Jilka, Peter O Jenkins, Gregory Scott, Lucia M Li, Nikolaos Gorgoraptis, David Baxter, David J Sharp, Anthony P Goldstone

**Supplementary Figures**

Supplementary Figure 1. Relationship between serum vitamin D and PTH concentrations

**Supplementary Tables**

Supplementary Table 1. Guidelines for definition of vitamin D status

Supplementary Table 2. Summary of meta-analyses of association studies between vitamin D status and depression

Supplementary Table 3. Summary of meta-analyses of intervention studies with vitamin D supplementation and depression

Supplementary Table 4. Demographics in outcome variable sub-groups

Supplementary Table 5. Correlations between vitamin D concentration and cognition, symptoms and quality of life

**SUPPLEMENTARY FIGURES**

**
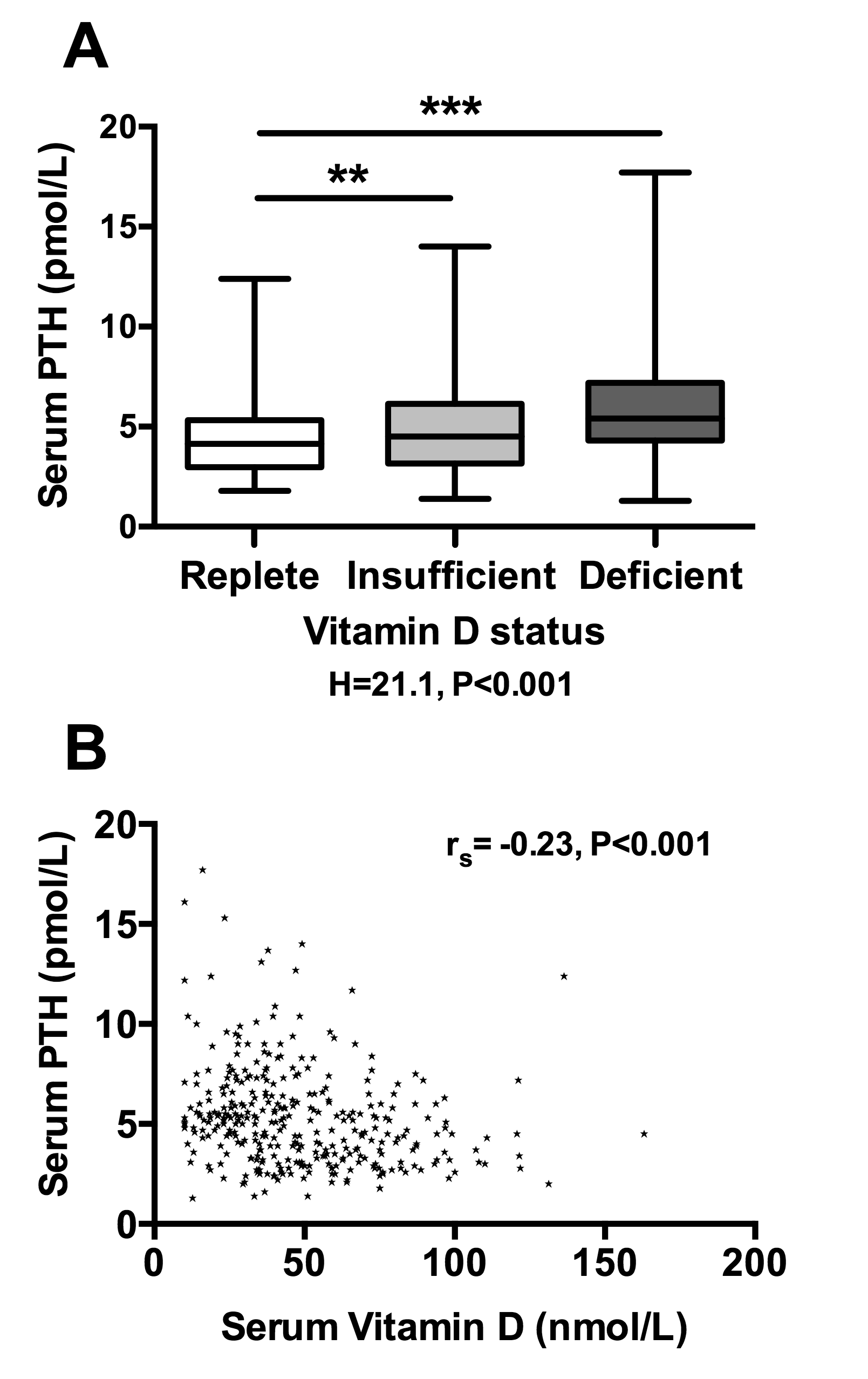
Supplementary Figure 1. Relationship between serum vitamin D and PTH concentrations**

Relationships between serum PTH concentration and (A) vitamin D status (box plots indicate median, interquartile range and range) and (B) serum vitamin D concentrations (both n=337). Statistical results from (A) Kruskal-Wallis one way ANOVA test: **P<0.01; , ***P<0.001; (B) r_s_ indicates Spearman's correlation coefficient; the relationship between serum vitamin D and PTH concentrations was not altered by season or skin color (no significant difference in slopes P=0.17 and P=0.53 respectively).

**SUPPLEMENTARY TABLES**

**Supplementary Table 1.** **Guidelines for definition of vitamin D status**

To convert from nmol/L to ng/mL divide by 2.496.

The North America Institute Of Medicine (IOM) and USA Endocrine Society guidelines are derived from two systematic reviews looking at the relationship between vitamin D status and bone health. The Imperial College Healthcare NHS Trust guidelines were generated from a literature review, incorporating values obtained from the particular immunoassay used, and lay between the IOM and Endocrine Society guidelines.

**^a^** Francis R, Aspray T, Fraser W, Gittoes N, Javaid K, Macdonald H, Patel S, Selby P, Tanna N, Bowring C. *Vitamin D and Bone Health: A Practical Clinical Guideline for Patient Management.* [Online] Available from: www.nos.org.uk/document.doc?id=1352 [Accessed 01/05/15].

**^b^** Holick MF, Binkley NC, Bischoff-Ferrari HA, Gordon CM, Hanley DA, Heaney RP, Murad MH, Weaver CM, Endocrine Society (2011). Evaluation, treatment, and prevention of vitamin D deficiency: an Endocrine Society clinical practice guideline. *J Clin Endocrinol Metab*, **96**,1911-1930.

**^c^** Tan T. ICHNT Guideline on Vitamin D Replacement in Adult Patients. Imperial College Healthcare NHS Trust. Approved November 2010

**Supplementary Table 2. Summary of meta-analyses of association studies between vitamin D status and depression**

^a^ Anglin RE, Samaan Z, Walter SD, McDonald SD (2013). Vitamin D deficiency and depression in adults: systematic review and meta-analysis. *Br J Psych*, **202**,100-107.

^b^ Ju SY, Lee YJ, Jeong SN (2013). Serum 25-hydroxyvitamin D levels and the risk of depression: a systematic review and meta-analysis. *J Nutr Health Aging*, **17**,447-455.

Abbreviations: CES-D, Center for Epidemiologic Studies Depression Scale; DSM, Diagnostic and Statistical Manual of Mental Disorders; F, female; GDS, Geriatric Depression Scale; ICD, International Classification of Diseases; M, male; N/A, not available; OR, odds ratio; SMD, standardised mean difference; UK, United Kingdom; USA, United States of America

**Supplementary Table 3. Summary of meta-analyses of intervention studies with vitamin D supplementation and depression**

^a^ Gowda U, Mutowo MP, Smith BJ, Wluka AE, Renzaho AMN (2015). Vitamin D supplementation to reduce depression in adults: meta-analysis of randomised controlled trials. *Nutrition*, **31**,421-429.

^b^ Li G, Mbuagbaw L, Samaan Z, Falavigna M, Zhang S, Adachi JD, Cheng J, Papaioannou A, Thabane L (2013). Efficacy of vitamin D supplementation in depression in adults: a systematic review. *J Clin Endo Metab*, **99**,757-767.

^c^ Shaffer JA, Edmondson D, Wasson LT, Falzon L, Homma K, Ezeokoli N, Li P, Davidson KW (2014). Vitamin D supplementation for depressive symptoms: a systematic review and meta-analysis of randomized controlled trials. *Psychosom Med*, **76**,190-6.

^d^ Spedding S (2014). Vitamin D and depression: a systematic review and meta-analysis comparing studies with and without biological flaws. *Nutrients*, **6**,1501-1518.

Abbreviations: BDI, Beck Depression Inventory; DSM, Diagnostic and Statistical Manual of Mental Disorders; F, females; GDS, Geriatric Depression Scale; HDRS, Hamilton Depression Rating Scale; M, males; SMD, standardised mean difference; UK, United Kingdom; USA, United States of America**.**

**Supplementary Table 4. Demographics in outcome variable** **sub-groups**

As particular assessments that were taken more than 31 days apart from blood sampling were excluded, the population for each assessment sub-group was slightly different. However, demographics were similar between each sub-group and the whole cohort.

Values stated as median [interquartile range] (min-max) or n (%) for categorical variables

Abbreviations: ACE-R, Addenbrooke's Cognitive Examination Revised; BDI-II, Beck Depression Inventory II; BMI, body mass index; NHP, Nottingham Health Profile; PSQI, Pittsburgh Sleep Quality Index; SF-36, Short Form 36; TBI, traumatic brain Injury

**Supplementary Table 5. Correlations between serum vitamin D concentration and cognition, symptoms and quality of life**

Statistical results from Spearman's correlation, except for ^a^ partial correlation when controlling for covariates.

P<0.006 used as significant for SF-36 after Bonferroni correction

P<0.008 used as significant for NHP after Bonferroni correction

*Abbreviations:* ACE-R, Addenbrooke's Cognitive Examination Revised; BDI-II, Beck Depression Inventory II; NHP, Nottingham Health Profile; PSQI, Pittsburgh Sleep Quality Index; PTA, post traumatic amnesia; QoL, quality of life; SF-36, Short Form 36; TBI, traumatic brain Injury
